# From Jumping Gene to Cancer: Revisiting the Role of JTB Protein

**DOI:** 10.3390/biomedicines13071705

**Published:** 2025-07-12

**Authors:** Taniya M. Jayaweera, Madhuri Jayathirtha, Krishan Weraduwage, Petra Kraus, Costel C. Darie, Anca-Narcisa Neagu

**Affiliations:** 1 Biochemistry & Proteomics Laboratories, Department of Chemistry and Biochemistry, Clarkson University, Potsdam, NY 13699, USA; jayawetm@clarkson.edu (T.M.J.); jayathm@clarkson.edu (M.J.); weraduk@clarkson.edu (K.W.); 2 Department of Biology, Clarkson University, Potsdam, NY 13699, USA; pkraus@clarkson.edu; 3 Laboratory of Animal Histology, Faculty of Biology, “Alexandru Ioan Cuza” University of Iași, Carol I bvd. 20A, 700505 Iasi, Romania

**Keywords:** jumping translocations (JTs), jumping translocation breakpoint (JTB) protein, tumorigenesis, biomarkers

## Abstract

Jumping translocations (JTs) are rare chromosomal abnormalities that play a crucial role in the pathogenesis of various cancer types. These rearrangements, especially those involving chromosome 1q, are frequently associated with tumor progression, therapeutic resistance, and poor prognosis. One gene of particular interest, human Jumping Translocation Breakpoint (*JTB*), has been identified at the site of translocation breakpoints and exhibits complex, context-dependent roles in cancer biology. JTB protein functions as a pivotal regulator in mitosis, chromosomal segregation, apoptosis, and cellular metabolism. It is functionally linked with the chromosomal passenger complex (CPC) and is implicated in processes such as epithelial–mesenchymal transition (EMT), immune evasion, and therapy resistance, especially in breast and prostate cancers. Advances in genomic, transcriptomic, and proteomic research have highlighted the significant potential of JTB as a diagnostic biomarker and a target for therapeutic interventions. This review underscores the dual role of JTB as both a tumor suppressor and oncogene, depending on the cellular context, and advocates for its continued investigation at the genomic, transcriptomic, and proteomic levels. Understanding JTB’s multifaceted contributions to tumor biology may pave the way for novel biomarkers and targeted treatments in cancer management.

## 1. Introduction

Chromosomal instability is a hallmark of cancer, frequently contributing to tumor initiation, progression, and resistance to therapy [1]. Among the diverse spectrum of chromosomal abnormalities, jumping translocations (JTs) represent a particularly rare and complex form of genomic rearrangement [2,3,4,5]. Characterized by the nonreciprocal translocation of a duplicated chromosomal segment from a single donor chromosome to multiple recipient chromosomes, JTs result in copy number alterations and disrupt genomic integrity across a variety of cell types. Although rare, these events have been documented in both constitutional and acquired forms and are notably recurrent in hematological malignancies, particularly myeloid cancers and multiple myeloma (MM) [3,4,6]. The preferential involvement of the 1q chromosomal region, especially 1q21, in these rearrangements underscores its potential role in driving malignant transformation [7].

Recent studies have increasingly highlighted the epigenetic and structural mechanisms behind JT formation, implicating DNA demethylation, chromatin decondensation, and telomere dysfunction as central factors in this process [8]. Furthermore, JTs appear to interfere with tumor suppressor gene function and contribute to clonal evolution, ultimately leading to poor clinical outcomes in affected patients. Among the genes located within the frequently rearranged 1q21 region is the *Jumping Translocation Breakpoint* (*JTB*) gene, which is a relatively underexplored gene that was initially identified in the context of JTs.

The *JTB* gene, also known as *Prostate Androgen-Regulated* (*PAR*), encodes an evolutionary conserved transmembrane protein with emerging roles in cell cycle control, mitochondrial function, and apoptosis [9,10,11]. JTB is dynamically expressed during mitosis and interacts with key regulators, such as Aurora kinases A and B (AURKA and AURKB), implicating it in processes that are crucial for genomic stability [10]. While *JTB* dysregulation—either overexpression or silencing—has been observed across various cancer types, including breast cancer (BC), prostate cancer (PCa), and MM, its precise role remains ambiguous. Intriguingly, proteomics-based studies have revealed that JTB may act as a context-dependent oncogene or tumor suppressor, depending on the cellular environment and regulatory cues [12].

JTB can be explored across genomic, transcriptomic, and proteomic levels, providing a multidimensional understanding of its role in cancer biology and reinforcing its potential as a comprehensive biomarker in translational research. *JTB* emerges as a gene of significant interest in understanding the oncogenic potential of JTs. This review synthesizes current knowledge on the genomic, molecular, and proteomic aspects of JTB, its role in cancer biology, and its potential value as a biomarker and therapeutic target. By exploring the intricate relationship among JTs, 1q21 amplification, and JTB protein functions, we aim to provide a comprehensive overview of these transcripts and isoproteins within the broader context of cancer genomics and precision medicine. Given its frequent dysregulation in various malignancies and its involvement in key oncogenic pathways, JTB protein holds promise as a diagnostic and prognostic biomarker, with potential utility in guiding personalized therapeutic strategies in clinical oncology settings [12,13,14,15,16,17].

## 2. Jumping Translocations (JTs) in Cancer: Mechanisms and Clinical Implications

Jumping translocations (JTs) represent rare and atypical chromosomal arrangements, either constitutional or acquired, that are classified as cytogenetic abnormalities. These events involve nonreciprocal translocations of the same duplicated chromosomal segment from a single donor chromosome to two or more different recipient chromosomes, often occurring in different cells [2,3,4,5]. This process typically results in copy number gains of the translocated fragment, along with potential losses in segments from the recipient chromosomes [6]. The donor fragment may integrate into the telomeric or interstitial regions of recipient chromosomes, giving rise to varied JT patterns [6]. Interestingly, breakpoints in JTs are non-randomly distributed, with a preference for the pericentromeric and telomeric regions of chromosomes [2]. Several mechanisms have been proposed to underlie JT formation, including viral infection, chromosomal instability, the decondensation of pericentromeric heterochromatin, telomere shortening, and aberrant recombination between telomeric repeat sequences and interstitial telomeric sequences [6].

In the study by Padilla-Nash et al. (2001), spectral karyotyping, combined with fluorescence in situ hybridization (FISH), was employed to examine ten solid tumor-derived cell lines originating from bladder, prostate, breast, cervical, and pancreatic carcinomas [18]. A total of 188 jumping translocations (JTs) and sequential jumping translocations (SJTs) were detected across these samples. Each cell line demonstrated multiple occurrences of both JTs and SJTs, leading to recurrent, unbalanced translocations involving entire chromosome arms, most notably 5p, 14q, 15q, 20q, and 21q [18]. Notably, 60% of the JT breakpoints were localized within centromeric or pericentromeric regions of the recipient chromosomes, whereas only 12% were situated near telomeric ends. Many breakpoints on both donor and recipient chromosomes coincided with fragile genomic sites and integration loci for human DNA viruses, indicating a possible mechanistic relationship [18]. Within each tumor cell line, the presence of JTs appeared to promote clonal evolution by enabling the acquisition of extra copies of specific chromosomal segments. These frequently included key oncogenes, such as *MYC*, the tyrosine-protein kinase *ABL*, and human epidermal growth factor receptor 2 (*HER2/neu*), which contribute to tumor-specific genomic imbalances that may drive cancer progression [18].

Although JTs are most frequently observed as acquired chromosomal abnormalities in hematologic malignancies, constitutional JTs remain exceptionally rare [19]. In a study by Lee et al. (2010), two such constitutional JTs were reported in chorionic villi samples from products of conception. In the first case, chromosome 18 acted as a donor, with fragments translocated to chromosomes 1, 13, 15, 18, and 21. In the second case, chromosome 22 was the donor, while chromosomes 1 and 9 served as recipients. Both events were de novo, indicating that they occurred spontaneously rather than being inherited [19]. The breakpoints were primarily located in the centromeric, pericentromeric, or telomeric regions. Although normal cell lines were also present in both cases, these findings support previous evidence that the genomic instability caused by JTs and the resulting chromosomal imbalances likely contributed to early pregnancy loss [19].

Jumping translocations involving chromosome 1q (1q-JT) have been proposed as potential biomarkers in myeloid malignancies due to their associations with recurrent mutations in key genes related to DNA repair, spliceosome assembly, and epigenetic regulation, all of which contribute to poor clinical outcomes [5]. These translocations reflect a state of chromosomal instability, which is induced by epigenetic alterations and shortened telomeres and linked with an increased risk of progression from myelodysplastic syndromes (MDS) to acute myeloid leukemia (AML), often correlating with an unfavorable poor prognosis [20]. In cases that progress from MDS to AML, approximately 82% of these fusions are thought to occur in the telomeric regions of either the p or q arms, while the remaining 18% are located in the pericentric or interstitial areas [20]. Contributing factors to the emergence of JTs include hypomethylation at the pericentromeric region of the 1q donor and telomere attrition in recipient chromosomes [20].

Chromosome 1 in region 2, band 1 (1q21), in particular, is recurrently altered across numerous cancer types [21]. Hatakeyama et al. (1999) described the *JTB* gene within this region, showing its fusion with telomeric sequences in JT events [21]. The amplification of 1q21, which is associated with the overexpression of oncogenes within the 1q21 amplicon, is also frequently detected in hepatocellular carcinoma (HCC) [22]. The overexpression of these oncogenes could be correlated with cell cycle promotion and apoptosis inhibition [22]. In non-Hodgkin’s lymphoma (NHL), approximately 30% of 1q breakpoints affect the proximal 1q21 area, with involvement noted in follicular lymphoma, diffuse large B-cell lymphoma, and diffuse small cell lymphoma [23]. Furthermore, breakpoints at 1q21 have been implicated in both reproductive disorders, such as pregestational infertility [6], and hematologic malignancies like MDS and AML, where breakpoints are commonly localized to the pericentric region of 1q21 [20]. The 1q21 region has been linked to the pathophysiological processes that underlie disease progression and drug resistance in MM [24].

In cases that progressed from MDS to AML, the most common telomeric fusion partners for 1q-JTs were chromosomes 15p, 22p, 21p, 14p, 13p, Yq, 17q, 18q, and 21q [20]. Among the pericentric and interstitial fusion sites, the most frequent were 16q11.2–q12, 7q11, and 20q12–q13.1 [20]. Notably, in three-quarters of analyzed cases, the 1q segment was found to be translocated to the short-arm telomeric regions of at least one acrocentric chromosome, specifically chromosomes 13, 14, 15, 21, or 22 [20]. In multiple melanoma (MM), recurrent whole-arm translocations of 1q were observed with several partner chromosomes, including 5, 8, 12, 14, 15, 16, 17, 19, 21, and 22 [25].

In summary, jumping translocations (JTs) represent a rare but impactful form of chromosomal instability that contributes to the development and progression of a wide range of malignancies, including both hematologic and myeloid cancers, as well as solid tumors. Their non-random breakpoint distribution, frequent involvement of oncogene-rich chromosomal regions, and capacity to induce copy number alterations underscore their oncogenic potential. The predominance of 1q-JTs, especially those involving the 1q12-q21 region, highlights a recurrent pattern associated with disease progression, treatment resistance, and poor clinical outcomes. Moreover, recent insights into the epigenetic and molecular mechanisms that drive JT formation—such as chromatin remodeling, DNA methylation changes, and viral integration—reveal a complex interplay between genomic architecture and tumor evolution. While constitutional JTs remain uncommon, their presence in early developmental stages further emphasizes the disruptive potential of these rearrangements. As research continues to uncover the genomic and epigenomic contexts of JTs, their role as both diagnostic biomarkers and therapeutic targets may become increasingly relevant in precision oncology.

## 3. From Gene to Protein: Multifaceted Roles of JTB and Implications in Cancer

### 3.1. Discovery and Genomic Context of JTB

Approximately 26 years ago, Hatakeyama et al. (1999) identified a novel human gene, *Jumping Translocation Breakpoint* (*JTB*), located at chromosome 1q21, which was found to fuse with telomeric repeats of recipient chromosomes in a case of jumping translocation (JT) [21]. This gene is described to be situated within the epidermal differentiation complex (EDC) [26] and is functionally associated with the chromosomal passenger proteins/complex (CPP/CPC), which are key regulators of mitosis [10]. The chromosomal passenger complex (CPC) is a key protein assembly that orchestrates critical events during cell division [27]. It consists of AURKB, along with inner centromere protein (INCENP), borealin (BOR), and survivin (BIRC5). Throughout mitosis, the CPC dynamically relocates to distinct cellular structures, where it ensures accurate chromosome–spindle attachments, activates the spindle assembly checkpoint to monitor chromosome alignment, and regulates the formation and function of the contractile apparatus, which is essential for successful cytokinesis. Originally thought to be a passive chromosome-associated complex, the CPC is now understood to be a pivotal regulator of mitotic progression.

Based on data from the Gene Set Enrichment Analysis (GSEA) platform (accessed on 23 April 2025), the chromosomal region 1q21 in humans contains a total of 404 annotated genes [28]. Genomic alterations that involve the gain or amplification of 1q21 (1q21+) are frequently linked to the dysregulation of oncogenes and cancer-related pathways [29]. Approximately 40% of MM patients exhibit 1q21+, which is a cytogenetic abnormality associated with increased tumor burden, more extensive end-organ damage, the co-occurrence of other high-risk alterations, and a greater likelihood of drug resistance, rapid disease progression, and reduced overall survival compared to patients without this aberration [29]. As a result, current research and clinical trials have increasingly emphasized the role of 1q21+ in therapeutic response and personalized treatment strategies [29].

JTs are commonly observed in both solid tumors and hematologic malignancies, including acute leukemia, MM, and non-Hodgkin lymphoma. In the majority of these cases, chromosomal segments distal to the 1q21 region are translocated [30]. These unbalanced translocations often result in the acquisition of additional copies of chromosome 1q, which have been strongly linked to tumor progression and aggressiveness [30]. Nagai et al. (2010) described a case in which a JT involving 1q21 emerged during the complete remission of AML and persisted for over 14 years without any evidence of malignant transformation [30]. The EDC is a gene cluster that is generally associated with human skin diseases, such as psoriasis, atopic dermatitis, and hyperkeratosis; however, it has been linked to numerous cancers, including skin, gastric, colorectal (CRC), lung, ovarian, and renal carcinomas [31]. Genes within the EDC primarily play a key role in epidermal development through mechanisms that involve epigenetic modifiers or unique chromatin remodeling factors [32]. However, in cancer, skin disorders, and other conditions, disruptions in these epigenetic mechanisms can lead to aberrant cell proliferation and differentiation [32]. The EDC genes are located within a 2 Mb region of human chromosome 1q21 and encode structural and regulatory proteins that are crucial for the terminal differentiation of keratinocytes and *stratum corneum* properties in mammals, reptiles, and birds [33,34,35]. The EDC was later identified on chromosome 3q of the mouse [35]. On the other hand, the CPC, which is composed of AURKB, INCENP, BOR, and BIRC5, with which JTB appears to be functionally associated, plays a critical role in ensuring accurate chromosomal alignment, segregation, and cytokinesis during mitosis and has been identified as a potential target for cancer therapy [36,37]. The CPC is also evolutionarily conserved and essential for accurate genome transmission during cell division, while disruptions in this complex lead to chromosomal instability and aneuploidy, which are well-established hallmarks of cancer [38].

### 3.2. Structural Features and JTB Molecular Interactions

**Figure 1:**
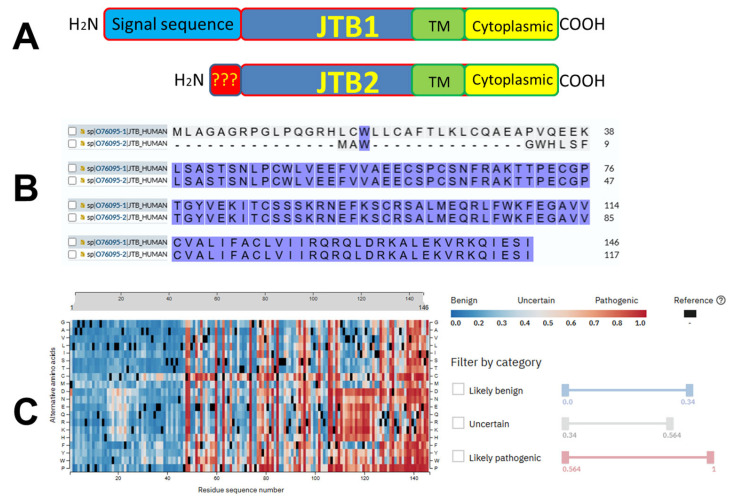
(**A**) Schematic of JTB1 and JTB2 highlighting the similarities and differences between the two isoforms. (**B**) Alignment of the amino acid sequence of the two JTB isoforms. (**C**) Alpha missense pathogenicity map for JTB1 (and JTB2).

The human *JTB* gene encodes a 146-amino acid transmembrane protein with a molecular weight of 16.358 kDa. It is evolutionarily conserved across diverse eukaryotic species, from nematodes to humans (UniProt ID: O76095) [9,11,21]. *JTB* orthologs with conserved gene structures have been identified in several primate species, including the Western lowland gorilla (*Gorilla gorilla gorilla*), the chimpanzee (*Pan troglodytes*), and the bonobo (*Pan paniscus*) (UniProt ID: O76095). In chimpanzees, two isoforms of the JTB protein have been described: isoform 1, which consists of 146 amino acids, and isoform 2, which is shorter, consisting of 117 amino acids (UniProt ID: O76095). The first isoform of JTB, JTB1, contains a signal sequence, a cysteine-rich extracellular domain, a transmembrane domain, and a cytoplasmic domain. The second isoform of JTB (JTB2) has either a similar or identical amino acid sequence, except in the N-terminal region, where the signal sequence is missing (Figure 1A). A third isoform is also suspected: a truncated form of JTB1 that is devoid of the cytoplasmic and transmembrane domains and is most likely produced via JTB translocation.

Rousseau et al. (2012) showed that JTB functions as an orphan receptor, and translocations often occur at the *JTB* genomic locus [11]. These events lead to multiple copies of a truncated *JTB* gene, potentially encoding a soluble, secreted ectodomain [11]. In this study, the authors report the structure of the N-terminal ectodomain of human JTB, which adopts a unique folding pattern resembling a novel form of a three-stranded antiparallel β-meander. The overall architecture of JTB shares structural features with the midkine/pleiotrophin family, particularly in the conserved arrangement of disulfide bonds. This small, cysteine-rich domain highlights the potential role of JTB in mediating interactions with other proteins or components of the extracellular matrix (ECM), offering insights into its still poorly understood biological functions [11]. The structural features suggest a possible extracellular role, potentially involving interactions with ECM components or other proteins. Furthermore, JTB protein has been shown to be dynamic during the cell cycle, peaking during the G2 and M phases. JTB interacts with mitotic regulators such as AURKA and AURKB, implicating JTB in spindle assembly, chromosome segregation, and cytokinesis [10].

**Figure 2:**
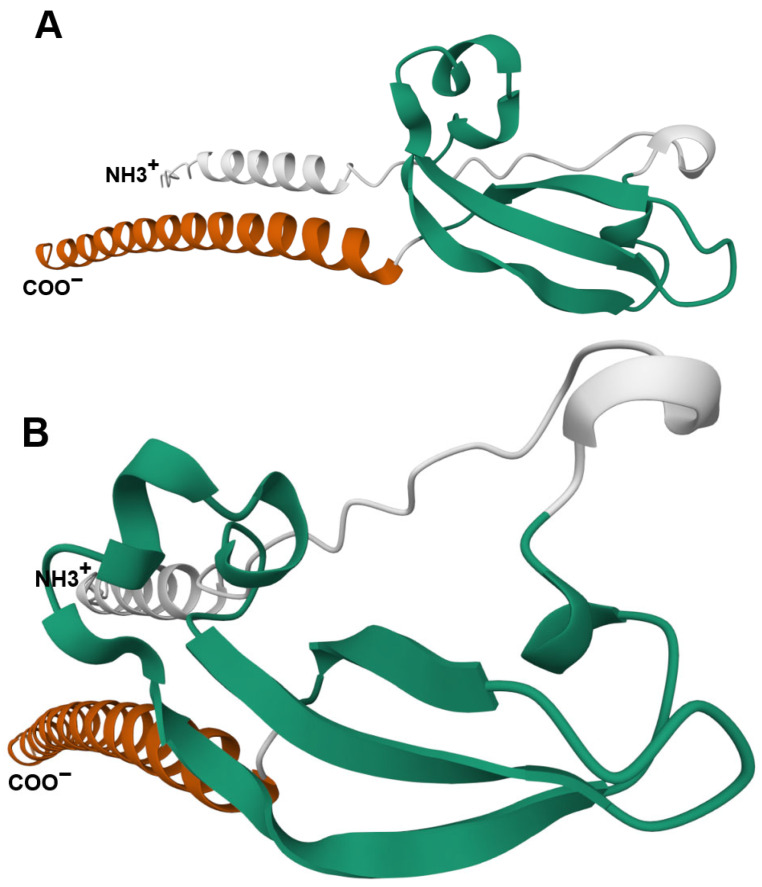

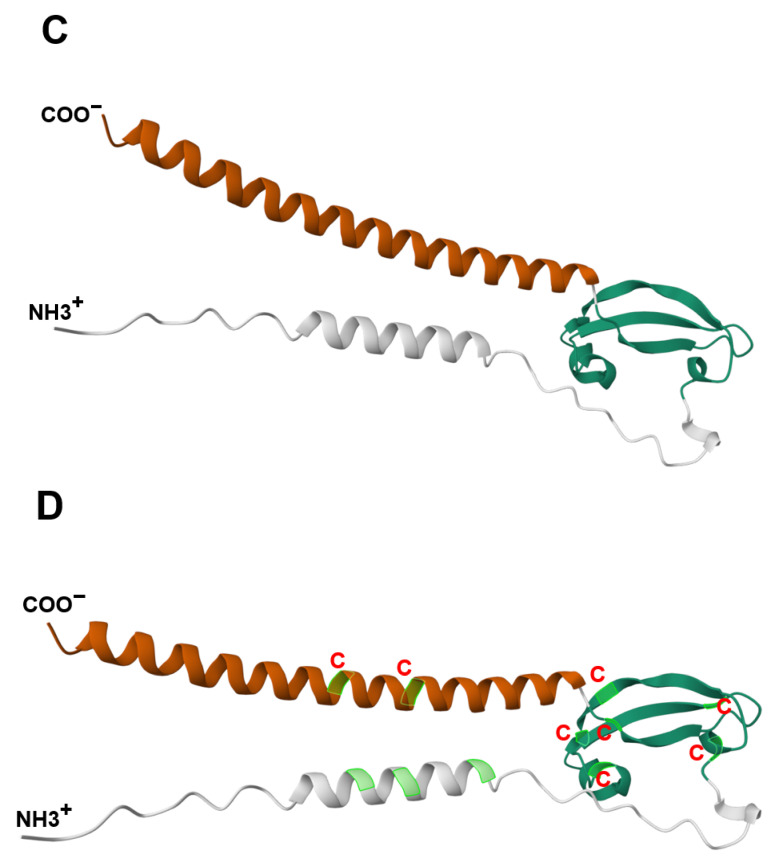
Three-dimensional structure of JTB1. The N- and C-termini of this protein are indicated. The cysteine residues (C) within the cysteine-rich region and the intracellular cytoplasmatic region are indicated. The structure was obtained using the prediction model generated by the Alphafold program [39]. (**A**–**D**) represents the same protein, viewed from different angles.

**Figure 3:**
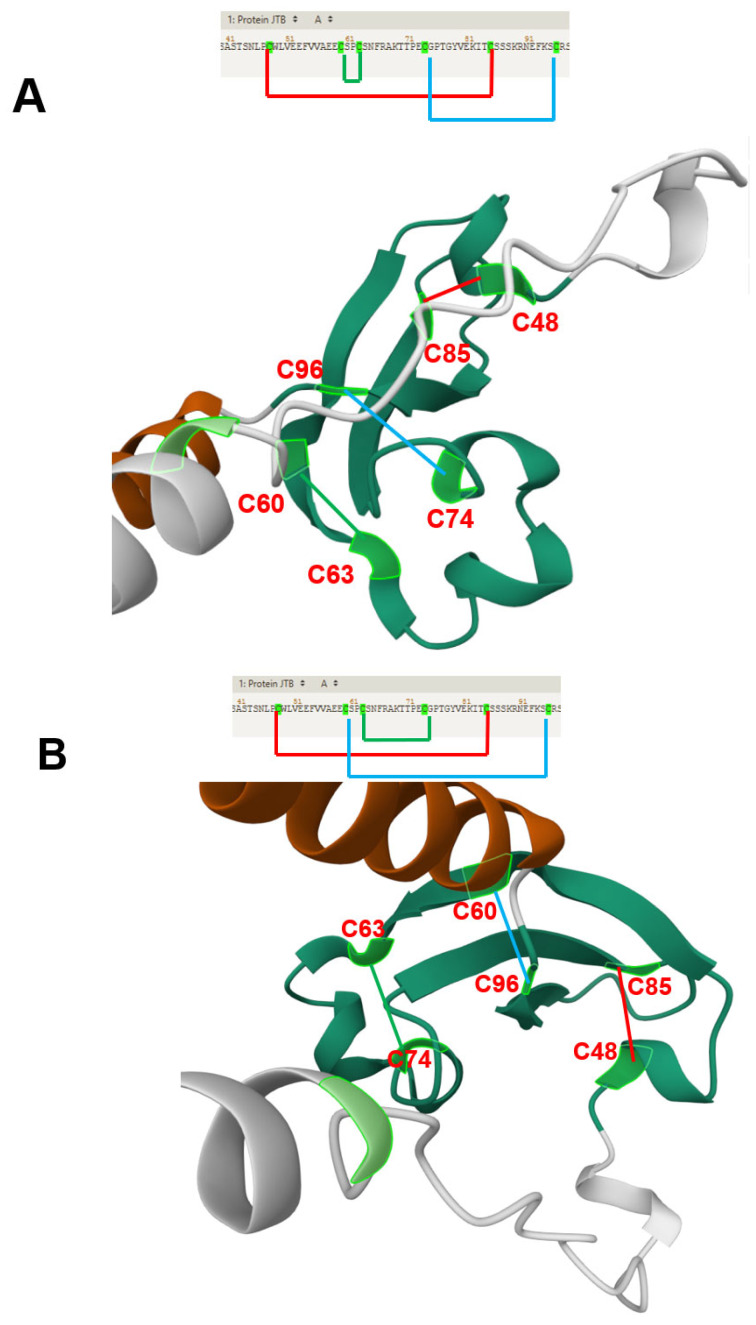

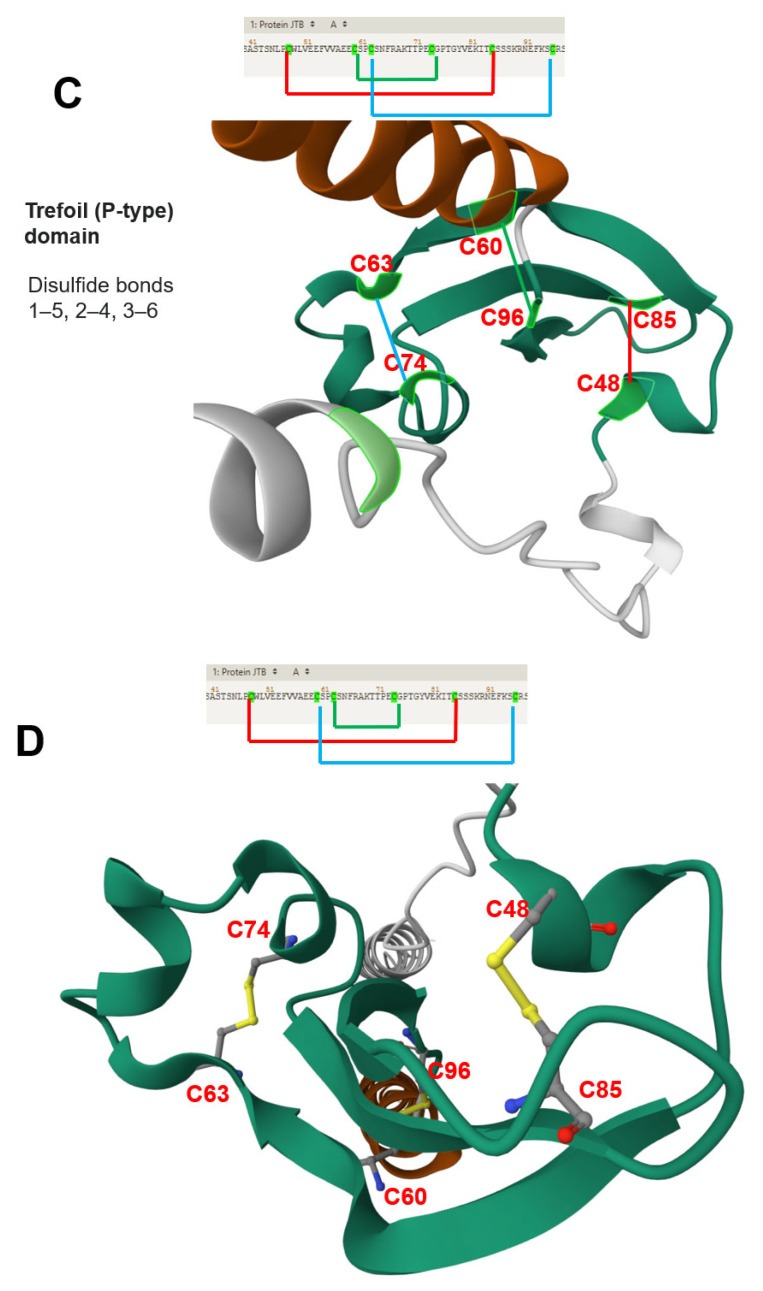
(**A**–**C**) The potential cysteine connectivity within JTB1 based on the distance between the cysteine residues within the three-dimensional structure of the cysteine-rich domain, as seen from different vantage points. (**D**) The cysteine connectivities as predicted by the Alphafold model (and confirmed by the NMR structure). Cysteine residues (C) are indicated and their position within the protein is also shown (i.e., C48 is amino acid 48 within protein, from the N-terminus).

The sequence alignment of the two JTB isoproteins is shown in Figure 1B. An Alphafold pathogeny map analysis program suggests that the most pathogenic region of these JTB isoproteins is in the cytoplasmic C-terminal region. In addition, the six conserved cysteine residues from the cysteine-rich region (part of the extracellular domain) are also considered pathogenic (Figure 1C) [39]. The structure of the canonical JTB (JTB1) is shown in Figure 2. As observed, according to the Alphafold prediction, the N- and C- terminal regions form alpha helixes, while the cysteine rich region is mostly a beta-sheet structure. The conserved cysteine residues within JTB (both JTB1 and JTB2) are shown in Figure 2D. Two such cysteines are in the cytoplasmic C-terminal region, which are most likely reduced, due to the highly reducing intracellular environment. The other six cysteines, all extracellular, are involved in disulfide bridges. A closer look at the Alphafold structures (Figure 3) suggest three possible disulfide bridges based on the close proximity of the cysteines to each other. One disulfide bridge option would be 1–5, 2–3, 4–6 (Figure 3A), the second option would be 1–5, 2–6, 3–4 (Figure 3B), and the third option would be 1–6, 2–4, 3–6 (Figure 3C). However, both Alphafold predictions were, in fact, 1–5, 2–6, 3–4, which is identical to the model determined by Rousseau et al. using NMR [11]. It is yet to be determined where the extracellular domain of JTB2 would be located, given that despite the presence of a transmembrane domain, it does not have a signal sequence.

### 3.3. Functional Implications in Normal and Malignant Cells

JTB protein is broadly expressed in normal human tissues; however, its expression is often dysregulated in various malignancies, exhibiting either over- or underexpression, depending on the specific tumor type [9]. Kanome et al. (2007) reported that JTB protein expression is frequently downregulated in tumors from different organs, suggesting its potential involvement in the neoplastic transformation process [7]. In contrast, Platica et al. (2011), as well as Rousseau et al. (2012), showed that JTB protein is overexpressed in many human tumors, including ovary, breast, lung, uterus, and colon cancer types, highlighting its potential as a therapeutic target [10,11]. Their research utilized breast cancer (BC) cell lines (MCF7 and T47D), as well as androgen-independent prostate cancer (PCa) cell lines (DU145 and LNCaP) [10].

Functional studies have demonstrated the oncogenic potential of JTB: when NIH3T3 fibroblasts were transfected with *JTB* cDNA, the cells exhibited enhanced growth in cultures, colony formation in soft agar, cell cycle acceleration (specifically shortened G1 and S phases), and tumor formation in SCID mice [40]. Furthermore, co-transfection with a 22-mer antisense oligonucleotide targeting *JTB* mRNA suppressed these tumorigenic behaviors, including the loss of anchorage-independent growth. These findings, which are supported by additional studies involving the DU145 PCa cell line transfected with antisense *JTB* cDNA, suggest that the *JTB* gene exhibits proto-oncogenic properties [9,40].

### 3.4. JTB and Mitotic Regulation

JTB demonstrates dynamic expression throughout the cell cycle, reaching its highest levels during the G2 and M phases [10]. Evidence suggests that JTB may function as an activator of AURKA [10], a mitotic serine/threonine kinase whose expression and activity are also cell cycle-dependent. AURKA is primarily localized at the centrosome during the G2 and M phases, where it plays a critical role in centrosome maturation and separation and spindle assembly [41]. Conversely, its expression is significantly reduced during the G1 and S phases [41]. In normal cells, AURKA becomes active from the G2 phase onward, contributing to key processes, such as centrosome duplication and bipolar spindle formation. Additionally, it helps maintain the structural integrity of the Golgi apparatus and is typically degraded via ubiquitin-mediated pathways following mitosis [41]. In malignant cells, AURKA exhibits a dual role [41]. Its overexpression in various tumor types enhances cell proliferation by modulating mitotic regulators such as protein phosphatase 1 (PP1), polo-like kinase 1 (PLK1), targeting protein for XKLP2 (TPX2), and large tumor suppressor 1/2 (LAST1/2). Moreover, AURKA also participates in the non-mitotic signaling pathways that facilitate tumor cell invasion, metastasis, and therapeutic resistance, including resistance to chemotherapy, radiotherapy, and immune-based treatments [41].

JTB’s subcellular localization also shifts dynamically throughout mitosis [10]. During prophase and metaphase, JTB is situated at the centrosomes, while in anaphase, it relocates to the spindle midzone, eventually accumulating at the midbody during telophase and cytokinesis [10]. A portion of JTB can also be detected in the cytoplasm during mitosis [10]. Furthermore, JTB has been shown to interact with Aurora B kinase (AURKB) and inner centromere protein (INCENP), thereby enhancing the AURKB-mediated phosphorylation of histone H3 [10]. Silencing JTB in DU145 PCa cells leads to several mitotic defects, including improper chromosome alignment and segregation, failed cytokinesis, increased polyploidy, elevated apoptosis, and mitotic abnormalities that contribute to genomic instability and tumor progression [10]. The inhibition of AURKA disrupts mitotic spindle formation, often resulting in a unipolar spindle with two centrosomes that fail to separate properly [41].

### 3.5. JTB Contributes to Neoplastic Transformation by Disrupting Mitochondrial Function

Additionally, JTB has been identified as a transforming growth factor beta-1 (TGF-β1)-inducible gene, which implies its role in cellular responses to this widely expressed cytokine [7]. TGF-β exhibits a dual role in cancer progression, in which it functions as a tumor suppressor in the early stages of malignancy but promotes tumorigenesis at later stages by enhancing cellular transformation, the epithelial-to-mesenchymal transition (EMT), invasion, and metastasis [42]. Functional studies have demonstrated that JTB undergoes N-terminal processing and primarily localizes to mitochondria [7]. When expressed in cells, JTB induces mitochondrial clustering around the nuclear periphery, swelling of individual mitochondria, and a significant reduction in mitochondrial membrane potential, resulting in mitochondrial dysfunction. These changes are associated with suppressed cell proliferation and increased resistance to TGF-β1-induced apoptosis. Notably, these effects depend on the proper N-terminal cleavage of the protein, as cleavage-resistant mutants do not exhibit these alterations. These findings suggest that structural or expression abnormalities of JTB may contribute to neoplastic transformation by disrupting mitochondrial function, leading to deregulated cell growth and/or survival [7].

### 3.6. JTB’s Role in Hematologic Malignancies

JTs have been linked to disease progression in hematologic cancers, particularly MDS, AML [8], and MM [3,4,6]. In myeloid malignancies, JTs are typically acquired as a late event, with a median onset of time to 24.9 month after diagnosis; these cases often present with features of myelodysplasia, and in all reported instances, the donor segment originated from chromosome 1 [4]. The formation of 1q-JTs appears to follow a multistep process, particularly during the progression from MDS to AML, offering a potential mechanistic framework for JT in leukemia development [6]. These chromosomal alterations are generally associated with poor treatment response, disease progression to AML, and reduced overall survival [4,5]. Most 1q-JTs involve the 1q12-q21 chromosomal region and have been documented in approximately 50 myeloid cancer cases, although mutation data are limited [5]. While other chromosomes, such as 3, 11, 15, and 21, have also served as donor chromosomes in myeloid malignancies, 1q remains the most prevalent [4,43]. Kondo et al. (2020) described a case involving a 1q-JT observed in MDS that originated from donor-derived umbilical cord blood cells [44]. This finding is particularly noteworthy, as donor cell-derived leukemia and MDS are uncommon complications following allogenic stem cell transplantation [44]. In chronic lymphocytic leukaemia (CLL), the most frequent JT breakpoint has been mapped to 17p11.2, with *TP53* gene loss observed in 88% of cases either prior to or concurrent with JT formation [3]. These alterations are considered recurrent in aggressive forms of CLL, contributing to complex karyotypes and associated with the loss of the tumor suppressor gene *TP53* [3].

A recent study by Lema Fernandez et al. (2024) investigated *SRSF2*-mutated myeloid neoplasms, including MM, and demonstrated that 1q-JTs arise following DNA demethylation and chromatin decondensation [8]. These rearrangements lead to the translocation of tri- or tetrasomic copies of 1q segments to multiple recipient chromosomes [8]. During disease progression, these cells exhibited a shift toward hypermethylation and the epigenetic involvement of the PI3K/AKT and MAPK signaling pathways, with AKT1 phosphorylation emerging as a hallmark of cancer advancements [8]. This highlights the epigenetic landscape as a powerful lens for studying repetitive DNA rearrangements and their roles in cancer evolution [8]. Lee et al. (2019) demonstrated that the formation of 1q jumping translocations (1qJTs) likely occurs through multiple sequential stages [6]. Their findings also suggest that the presence of 1qJT constitutes a particularly high-risk cytogenetic abnormality that is strongly associated with the progression of MDS to AML.

### 3.7. JTB in Prostate Cancer: Androgen Regulation and Therapeutic Implications

Furthermore, JTB expression was found to be significantly higher in PC3 cells, which is an androgen-independent PCa cell line that closely resembles the highly aggressive small cell neuroendocrine carcinoma (SCNC) subtype, compared to its expression in LNCaP cells, which represent an androgen-sensitive human prostate adenocarcinoma cell line [45]. Notably, siRNA-mediated silencing of the *JTB* gene in PC3 cells resulted in a reversion of their malignant phenotype, highlighting a potential role of JTB in driving tumor aggressiveness [45]. In conclusion, the *JTB* gene may act downstream of the androgen receptor (AR), given its role in promoting malignant proliferation in PCa cells [45]. These findings suggest that JTB could represent a promising therapeutic target, particularly in cases of androgen-independent PCa in which the AR signaling pathway is altered [45].

### 3.8. Proteomics-Based Characterization of JTB in Breast Cancer (BC)

Jayathirtha et al. [12,13,14,15,16] have provided evidence that the JTB protein plays a complex role in BC, functioning contextually as either an oncogene or tumor suppressor. Their proteomics-based investigations using the MCF7 BC cell line revealed that both the overexpression and downregulation of JTB significantly disrupt multiple cellular pathways and biological processes in BC cells. These authors employed a multi-platform proteomics strategy to investigate the role of JTB protein in BC, particularly using the MCF7 cell line model. Their integrated cellular proteomics-based approach combined sodium dodecyl sulfate–polyacrylamide gel electrophoresis (SDS-PAGE) [13,14], in-solution digestion [15], and two-dimensional polyacrylamide gel electrophoresis (2D-PAGE) [12,16], each coupled with nano-liquid chromatography tandem mass spectrometry (nLC-MS/MS). These complementary techniques enabled the identification of differentially expressed proteins (DEPs) associated with both JTB overexpression and JTB silencing, providing a multidimensional view of JTB’s impact on cellular pathways and biological processes. Importantly, the use of these different methods uncovered overlapping but also unique sets of proteins and pathways, revealing both pro-tumorigenic and tumor-suppressive roles of JTB, depending on its expression level. While JTB overexpression enhanced pathways that promote cell proliferation, migration, and resistance to apoptosis, JTB silencing similarly induced invasive and metabolically reprogrammed phenotypes, indicating that JTB dysregulation—either up or down—disrupts cellular homeostasis in favor of tumor progression. This triangulated proteomics approach not only reinforced the reproducibility and validity of findings across platforms but also provided a comprehensive systems-level understanding of JTB’s involvement in BC biology. By integrating data from diverse proteomic workflows, the authors demonstrated the utility of multi-method proteomics for uncovering complex oncogenic networks and identifying potential biomarkers and therapeutic targets.

Using SDS-PAGE coupled with nLC-MS/MS, Jayathirtha et al. (2022) revealed that the overexpression of JTB in MCF7 BC cells led to significant dysregulation in various cellular pathways, including mitotic spindle assembly, estrogen response, and EMT [13]. Key proteins related to cell division, cytoskeletal organization, estrogen response, lipid biogenesis, migration, and metastasis were upregulated. Furthermore, overexpressed JTB was associated with altered metabolic and stress response pathways, as well as resistance to cancer therapies. These findings highlight JTB’s potential contribution to tumorigenesis, particularly in regulating cell division, estrogen signaling, and cellular responses to environmental changes [13]. The downregulation of JTB in MCF7 BC cells leads to a more aggressive, invasive phenotype [14]. This shift is associated with the upregulation of proteins that promote actin cytoskeleton reorganization, EMT, enhanced cell motility, invasion, metabolic reprogramming, and immune evasion. The key pathways affected include glycolysis, fatty acid metabolism, cell cycle regulation, inflammatory signaling, and response to oxidative stress (OS) and hypoxia. Some downregulated proteins that normally suppress tumor progression or apoptosis are also reduced, further enhancing the tumorigenic potential. Overall, JTB downregulation drives MCF7 cells toward a phenotype characterized by enhanced proliferation, migration, invasion, and resistance to a hostile tumor microenvironment (TME) [14].

Another study based on in-solution proteomics conducted by Jayathirtha et al. (2022) also highlighted that JTB dysregulation, either overexpression or downregulation, in the MCF7 BC cell line significantly alters key biological processes, including EMT, cytoskeleton organization, metabolic reprogramming, and cellular proteostasis [15]. A proteomics analysis revealed that JTB influences mitochondrial function, OS response, apoptosis, and interferon signaling pathways. These findings again suggest that JTB contributes to the acquisition of a more aggressive cancer phenotype, despite MCF7’s typically non-invasive nature. Furthermore, overlapping results from both in-gel and in-solution proteomics approaches reinforce JTB’s association with enhanced proliferation, invasion, and metastatic potential, which are mediated through pathways such as EMT, mitotic spindle assembly, and fatty acid metabolism. Nevertheless, the presence of downregulated proteins with known antitumor roles in JTB-dysregulated conditions indicates a possible counterbalance that may moderate the oncogenic effects. Overall, JTB emerges as a potential biomarker and therapeutic target in breast cancer, warranting further investigation into its molecular mechanisms and interactions [15].

Jayathirtha et al. (2023) provided additional insights into the complex role of the JTB protein in BC, supporting its dual function as both a potential oncogene and tumor suppressor [12]. Using 2D-PAGE coupled with nLC-MS/MS, these authors identified the dysregulated proteins associated with JTB overexpression in MCF7 cells, many of which are involved in the EMT and other tumorigenic pathways. Notably, several downregulated proteins linked to JTB overexpression suggest tumor-suppressive functions, highlighting the context-dependent nature of JTB’s role. These results, when integrated with the previous SDS-PAGE and in-solution proteomics analyses, demonstrate the added value of a complementary proteomics approach to fully capture JTB-driven molecular changes [12]. Collectively, these data support the potential of JTB as a biomarker in BC and underscore the need for further mechanistic studies to elucidate its contribution to tumor initiation and progression [12]. Another study also conducted by Jayathirtha et al. (2023) supports the role of JTB protein as a potential tumor suppressor in BC [16]. Through 2D-PAGE, combined with nLC-MS/MS proteomics of MCF7 cells with JTB silencing, these authors identified the DEPs involved in key pro-tumorigenic pathways, including EMT, ERK/MAPK, PI3K/AKT, Wnt/β-catenin, and mTOR signaling. These DEPs are linked to enhanced proliferation, invasion, metabolic reprogramming, immune evasion, and the maintenance of stemness, indicating that JTB downregulation contributes to a more aggressive neoplastic phenotype. The consistency of these findings with the previous SDS-PAGE and in-solution proteomics analyses highlights the importance of a multi-platform approach for a comprehensive understanding of JTB-associated molecular mechanisms. Collectively, these data emphasize the potential of JTB as both a biomarker and a therapeutic target in BC, warranting further functional and clinical investigation [16]. Nevertheless, the paradoxical and multifaceted impact of JTB dysregulation highlights the necessity for further in vivo and mechanistic studies to determine its precise functional role in tumor initiation and progression.

### 3.9. JTB Expression Imbalance Promotes Malignant Phenotypes

**Figure 4:**
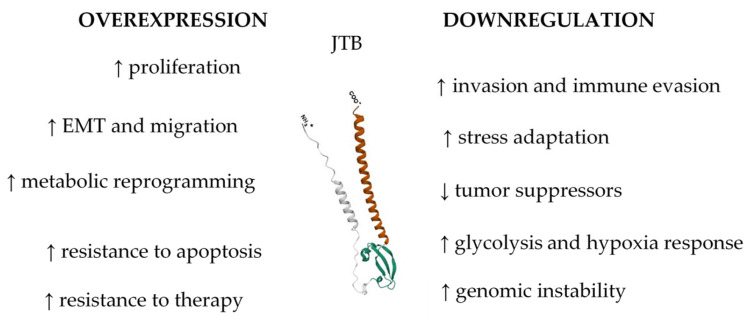
Role of JTB dysregulation in MCF7 breast cancer cell line.

Collectively, the findings from molecular, cellular, and proteomics-based studies converge on the conclusion that JTB protein is a complex and context-dependent regulator in BC, with the capacity to function both as an oncogene and a tumor suppressor. Across multiple experimental settings that involve the upregulation and downregulation of JTB in MCF7 BC cells [12,13,14,15,16,17], a consistent pattern of biological disruption was observed, indicating JTB’s pivotal role in modulating key tumorigenic processes (Figure 4). JTB overexpression was associated with increased cellular proliferation, enhanced metastatic potential, and phenotypic shifts toward more aggressive behavior, primarily through the activation of pathways related to cell division, hormone response, EMT, and metabolic reprogramming. On the other hand, JTB downregulation promoted similar malignant characteristics, suggesting that both the loss and gain of JTB function can destabilize cellular homeostasis and support tumor progression. These paradoxical findings reinforce the dualistic nature of JTB in cancer biology and highlight its capacity to participate in a wide range of cellular functions, including cell cycle regulation, apoptosis evasion, stress response, immune modulation, and cellular communication with the TME. The proteomics-based methodologies employed across these studies (SDS-PAGE, 2D-PAGE, and in-solution digestion) demonstrated the value of a multifaceted approach to fully characterize JTB’s molecular interactions and downstream effects. Overall, the collective results strongly support the potential of JTB as both a biomarker for BC diagnosis and prognosis, and as a target for therapeutic intervention. However, the context-dependent and multifactorial roles of JTB underscore the need for further in-depth mechanistic studies, particularly in vivo, to resolve its dual behavior and to evaluate its translational applicability in clinical oncology.

Key studies on JTB expression, function, and oncogenic potential are presented in Table 1.

## 4. Conclusions

Jumping translocations (JTs) represent rare yet significant chromosomal abnormalities that contribute to genomic instability across a range of malignancies. Rearrangements that involve chromosome 1q, especially the 1q21 region, are recurrent and strongly associated with disease progression, treatment resistance, and poor prognosis. The disruption and amplification of genes within this region, such as the *JTB* gene, underscore the potential oncogenic impact of JTs.

*JTB*, a gene originally identified at the breakpoint of a JT event, exhibits diverse and context-dependent functions in both normal and cancerous cells. Its dual role—acting either as a tumor suppressor or oncogene—appears to depend on the tumor type, expression level, and molecular context. Notably, JTB participates in critical cellular processes, such as mitotic regulation, chromosomal segregation, mitochondrial function, and apoptotic signaling. Moreover, it is functionally associated with the CPC, a mitotic regulator that is frequently implicated in cancer.

Recent advances in proteomics, alongside genomic and transcriptomic studies, have provided robust evidence for JTB’s role in modulating EMT, metabolism reprogramming, immune evasion, and therapeutic resistance, particularly in breast and prostate cancers. These findings highlight JTB’s potential utility, not only as a biomarker for disease progression and therapeutic stratification but also as a novel target for precision oncology. The elucidation of JTB’s mechanisms, particularly its involvement in key signaling pathways and protein–protein interactions (PPIs), can provide a clearer framework for understanding its potential in clinical diagnostics and therapeutic strategies. Additionally, MS coupled with 2D-PAGE offers a powerful approach to identifying JTB’s posttranslational modifications (PTMs) with high sensitivity and resolution, providing valuable insights into the functional implications of JTB in health and disease. The identification and characterization of PTMs are vital for uncovering the precise molecular mechanisms through which JTB influences cellular processes. To advance the clinical utility of JTB, further investigation into its tissue-specific expression patterns and interaction with other biomarkers could open new avenues for tailored treatments, particularly in complex diseases such as cancer.

In conclusion, the multifaceted nature of JTB in cancer biology, reflected in its genomic context, dynamic expression, molecular interactions, and context-specific functional effects, supports further investigation in clinical research. Integrating genomic, transcriptomic, and proteomic data will be essential to fully elucidate its oncogenic mechanisms, as well as to harness its potential in diagnostics, prognostics, and targeted therapies.

## Figures and Tables

**Table 1:** Summary of key studies on JTB expression, function, and oncogenic potential.

Authors	Year	Relevance	References
Hatakeyama et al.	1999	*JTB* gene was identified at 1q21 locus	[21]
		JTB is a transmembrane protein of 16.4 kDa evolutionary conserved across diverse eukaryotic species	
		The N-terminal hydrophobic region likely serves as a signal sequence for polypeptide secretion or membrane compartment recruitment, as it is processed and removed during this process	
		The C-terminal region is predicted to form the transmembrane domain, supporting the classification of JTB as a transmembrane protein	
		JT results in *JTB* truncation and a shortened protein variant that lacks the transmembrane and intracellular domains and is possibly secreted from cells	
		*JTB* is located in EDC	
Platica et al.	2000	*PAR* gene was isolated from LNCaP-OM androgen-resistant subline of PCa	[26]
		The complete sequence of the gene cDNA has 1029 nucleotides, with a continuous reading frame of 438 bases encoding for 146 amino acids	
		Amino acid sequence has motifs for myristoylation and phosphorylation by PKC	
		*PAR* gene was overexpressed in all PCa cell lines studied (LNCaP, DU145, PC3, and LNCaP-OM) compared to the normal prostatic tissue	
		PAR expression was higher in androgen-resistant prostate cancer lines (DU145, PC3, and LNCaP-OM) in comparison to androgen-sensitive cells (LNCaP)	
		PAR expression was downregulated by androgens in androgen-sensitive prostate cells, but not in the hormone-resistant cell lines	
		*PAR* gene is ubiquitously expressed in 29 normal studied tissues and overexpressed in most (67%) of their malignant counterparts	
		PAR expression was higher in the MCF7 and T47D BC cell lines, as well as in all primary breast tumors studied, compared to their normal tissue counterparts	
		PAR biological function is still unknown	
		Putative PAR involvement in basic cellular processes and malignant transformation	
Platica et al.	2001	PAR tends to be overexpressed in tumor cells	[40]
		PAR biological function is still unknown	
		Putative PAR implication in malignant transformation	
		Transfection of DU145 PCa cells with antisense *PAR* cDNA for PAR silencing led to decreased cell proliferation (arrest in G2/M phase) in tissue culture, low efficiency of colony formation in soft agar, and decreased tumor growth in SCID mice	
Platica et al.	2004	*PAR* is a 1038 bp gene located in chromosome 1 within EDC	[9]
		PAR is overexpressed in malignant tissues (proto-oncogene)	
		Transfection of NIH3T3 fibroblasts with *PAR* cDNA led to enhanced growth in culture, colony formation in soft agar, accelerated cell growth (shortened G1 and S phases), tumor formation in SCID mice	
		Transfection of NIH3T3 with 22-mer oligonucleotide in antisense orientation with PAR mRNA suppressed tumorigenic behaviors and abrogated colony formation in soft agar	
Xu et al.	2006	PAR expression was higher in PC3 PCa cells (more aggressive) than that in LNCaP cells	[45]
		DHT modulated PAR mRNA expression in LNCaP cells, and this effect was blocked by the AR antagonists	
		DHT did not affect PAR expression in PC3 cells	
		Reintroduction of AR into PC3 cells via stable transfection restored the androgen effect on PAR upregulation	
		siRNA transfection for PAR silencing in PC3 cells led to a reversal of the malignant phenotype	
		It is possible that PAR is downstream from the AR	
		PAR contributes to malignant proliferation in androgen-independent PCa cells	
		PAR could be a potential therapeutic target for androgen-independent PCa with AR signaling pathway alteration	
Kanome et al.	2007	JTB expression is suppressed in many cancers from different organs	[7]
		JTB plays a role in the neoplastic transformation of cells	
		JTB was isolated as a TGF-*β*1-inducible clone via differential screening	
		JTB may be processed at the N-terminus and is located mostly in mitochondria	
		JTB-induced clustering of mitochondria around the nuclear periphery and swelling of each mitochondrion	
		Mitochondria membrane potential was significantly reduced	
		JTB retarded the growth of the cells and conferred resistance to TGF-*β*1-induced apoptosis	
		These activities were dependent on the N-terminal processing and induced by wild-type JTB, but not by a mutant resistant to cleavage	
		Alterations in the structure or expression of JTB can lead to neoplastic changes in cells by disrupting mitochondrial function, resulting in uncontrolled cell growth and/or cell death	
Platica et al.	2011	PAR possesses oncogenic activity	[10]
		PAR has a dynamic expression throughout the cell cycle (lowest at G1/S, peaks in G2/M)	
		PAR’s subcellular localization shifts dynamically throughout mitosis	
		PAR is functionally related to CPP (mainly AURKA)	
		PAR changes AURKB activity	
		PAR silencing leads to defects during mitosis	
		PAR is overexpressed in cancer (OC, BC, lung, uterus, and colon cancer)	
		PAR is overexpressed in MCF7, T47D (BC cell lines), DU145, and LNCaP (PCa cell lines)	
		PAR degradation can occur by the ubiquitin–proteasome pathway	
Rousseau et al.	2012	JTB is an orphan receptor	[11]
		NMR analysis reveals a novel three-stranded antiparallel β-meander in the N-terminal ectodomain of JTB	
		JTB shows distant structural relation to midkine/pleiotrophin, especially in conserved disulfide bonds	
		Extracellular domain of JTB may be secreted and interact with proteins or ECM, suggesting roles in yet-undefined biological processes	
Jayathirtha et al.	2021	Study supports the hypothesis that JTB plays a role in tumorigenesis, particularly in BC, where it is frequently overexpressed	[17]
		Proteomic analysis of MCF7 cells with both upregulated and downregulated JTB expression emphasized in dysregulated proteins potentially linked to cancer-related pathways	
Jayathirtha et al.	2022	Cellular proteomics: MCF7 BC cells transfected with sense orientation of JTB cDNA for JTB overexpression; SDS-PAGE and nLC-MS/MS	[13]
		Overexpression of JTB in MCF7 BC cells led to significant dysregulation in various cellular pathways (mitotic spindle assembly, estrogen response, and EMT)	
		Key proteins related to cell division, cytoskeletal organization, estrogen response, lipid biogenesis, migration, and metastasis were upregulated	
		Overexpressed JTB was associated with altered metabolic and stress response pathways, as well as resistance to cancer therapies	
		JTB contributes to tumorigenesis, regulating cell division, estrogen signaling, and cellular responses to environmental changes	
Jayathirtha et al.	2022	Cellular proteomics: MCF7 BC cells transfected with shRNA plasmids for JTB silencing; SDS-PAGE and nLC-MS/MS	[14]
		Upregulation of proteins that promote actin cytoskeleton reorganization, EMT, cell motility, invasion, metabolic reprogramming, and immune evasion	
		Key pathways affected include glycolysis, FA metabolism, cell cycle regulation, inflammatory signaling, response to OS, and hypoxia	
		JTB downregulation drives MCF7 cells toward a phenotype characterized by enhanced proliferation, migration, invasion, and resistance to hostile TME	
Jayathirtha et al.	2022	Cellular proteomics: MCF7 BC cells transfected with sense orientation of the JTB cDNA for JTB upregulation and shRNA plasmid targeting the JTB mRNA for silencing; in-solution digestion-based cellular proteomics, nLC-MS/MS	[15]
		JTB dysregulation (both overexpression and downregulation) in the MCF7 BC cell line alters key biological processes (EMT, cytoskeleton organization, metabolic reprogramming, and cellular proteostasis)	
		JTB influences mitochondrial function, OS response, apoptosis, and interferon signaling pathways	
		JTB emerges as a potential biomarker and therapeutic target in BC, warranting further investigation into its molecular mechanisms and interactions	
Jayathirtha et al.	2023	Cellular proteomics: MCF7 cells transfected for JTB upregulation; 2D-PAGE coupled with LC-MS/MS	[12]
		JTB has a dual function as both a potential oncogene and a tumor suppressor, highlighting the context-dependent nature of JTB’s role	
		Data support the potential of JTB as a biomarker in BC and underscore the need for further mechanistic studies to elucidate its contribution to tumor initiation and progression	
Jayathirtha et al.	2023	Cellular proteomics: MCF7 cells transfected with shRNA plasmids for JTB downregulation; 2D-PAGE coupled with LC-MS/MS	[16]
		JTB interacting DEPs involved in key pro-tumorigenic pathways (EMT, ERK/MAPK, PI3K/AKT, Wnt/β-catenin, mTOR signaling)	
		DEPs are linked to enhanced proliferation, invasion, metabolic reprogramming, immune evasion, and maintenance of stemness, indicating that JTB silencing contributes to a more aggressive neoplastic phenotype	
		JTB protein may be a potential tumor suppressor in BC	
		Data emphasize the potential of JTB as both biomarker and therapeutic target in BC, warranting further functional and clinical investigation	
		Importance of a multi-platform proteomic approach for a comprehensive understanding of JTB-associated molecular mechanisms	

Abbreviations: AR—androgen receptor; AURKA—Aurora A protein kinase; AURKB—Aurora B protein kinase; BC—breast cancer; CPP—chromosomal passenger proteins; DEPs—differentially expressed proteins; DHT—dihydrotestosterone; EDC—Epidermal Differentiation Complex; EMT—epithelial-to-mesenchymal transition; FA—fatty acids; *JTB*-human Jumping Translocation Breakpoint gene; JT—jumping translocation; nLC-MS/MS—nano-liquid chromatography tandem mass spectrometry; NMR—nuclear magnetic resonance; OC—ovary cancer; OS—oxidative stress; PAR—prostate androgen-regulated; PCa—prostate cancer; PKC—protein kinase C; SCID—severe combined immunodeficient mice; SDS-PAGE—sodium dodecyl sulfate–polyacrylamide gel electrophoresis; TGF-β1—transforming growth factor beta; TME—tumor microenvironment; 2D-PAGE—two-dimensional polyacrylamide gel electrophoresis.

## Data Availability

Not applicable.

## Abbreviations

ABL	Abelson murine leukemia viral oncogene homolog
AML	acute myeloid leukemia
AURKA	Aurora kinase A
AURKB	Aurora kinase B
BC	breast cancer
BOR	borealin
CPC	chromosomal passenger complex
CRC	colorectal cancer
EDC	epidermal differentiation complex
EMT	epithelial–mesenchymal transition
FISH	fluorescence in situ hybridization
GSEA	Gene Set Enrichment Analysis
HCC	hepatocellular carcinoma
HER2/*neu*	human epidermal growth factor receptor 2
INCENP	inner centromere protein
JTB	jumping translocation breakpoint gene/protein
JT	jumping translocation
MDS	myelodysplastic syndrome
MM	multiple myeloma
MS	mass spectrometry
PAR	prostate androgen-regulated gene/protein
PCa	prostate cancer
PPI	protein–protein interaction
PTM	posttranslational modification
SJT	sequential jumping translocation
